# Pathogen-associated radiological and serum inflammatory findings in pediatric necrotizing pneumonia: a retrospective analysis

**DOI:** 10.3389/fped.2026.1749246

**Published:** 2026-04-10

**Authors:** Pei Tao, Shuai Hu, Liqun Li, Yinghong Fan, Zhigang Wang

**Affiliations:** Chengdu Women and Children’s Central Hospital, School of Medicine, University of Electronic Science and Technology of China, Sichuan, China

**Keywords:** community-acquired pneumonia, computed tomography, C-reactive protein, inflammatory markers, necrotizing pneumonia, pediatric, procalcitonin

## Abstract

**Background:**

Necrotizing pneumonia (NP) is a severe but relatively uncommon complication of pediatric community-acquired pneumonia. In recent years, increasing clinical attention has been directed toward pediatric NP. This study aimed to characterize the clinical features, chest CT findings, and serum inflammatory markers of pediatric NP, and to examine pathogen-associated differences in imaging patterns and inflammatory profiles.

**Materials and methods:**

We retrospectively reviewed 54 children diagnosed with NP between January 2018 and January 2024.Etiologic agents, chest CT findings, and serum inflammatory markers including white blood cell count (WBC), C-reactive protein (CRP), procalcitonin (PCT), lactate dehydrogenase, and D-dimer were analyzed. Pathogen-specific comparisons were restricted to single-pathogen cases to reduce classification bias. Mixed infections were analyzed descriptively and were not reassigned to any single-pathogen category.

**Results:**

Among 54 patients (mean age 6.29 ± 3.68 years), significant age-related differences in pathogen distribution were observed (*χ*^2^ = 18.7, *p* = 0.004). Bacterial pathogens were more common in younger children, whereas *Mycoplasma pneumoniae* predominated in older age groups. Bacterial prevalence showed a negative association with age (Spearman *ρ* = −0.82, *p* < 0.001), while *M. pneumoniae* prevalence showed a positive association (*ρ* = 0.79, 95% CI 0.65–0.88). Increasing age was inversely associated with complication severity (OR 0.54 per year increase, 95% CI 0.38–0.72). Identified pathogens included *Staphylococcus aureus* (*n* = 8), *Streptococcus pneumoniae* (*n* = 5), *Mycoplasma pneumoniae* (*n* = 28), adenovirus (*n* = 1), *Haemophilus influenzae* (*n* = 2), and mixed infections (*n* = 9), most commonly involving *M. pneumoniae* combined with bacterial or viral pathogens. Chest CT demonstrated consolidation with necrosis and cavitation across pathogen groups. *S. aureus* infections were associated with higher CRP levels [median 140 (IQR 71.8–200) mg/L] compared with *S. pneumoniae* [20.5 (8–200) mg/L] and *M. pneumoniae* [36 (11.5–85.64) mg/L; *p* < 0.01]. In contrast, *M. pneumoniae* infections showed lower PCT levels [0.16 (0.15–0.31) ng/L] than bacterial infections. No significant inter-group differences were found in WBC or D-dimer levels. Variations in arterial-phase CT attenuation were descriptively observed across pathogen groups.

**Conclusions:**

In this cohort, pediatric necrotizing pneumonia was associated with age-related variations in pathogen distribution, inflammatory markers, and chest CT characteristics. Elevated CRP levels were observed in *Staphylococcus aureus* infections, whereas lower PCT levels were noted in *Mycoplasma pneumoniae* cases. Variations in cavitary patterns were descriptively observed across pathogen groups. Given the retrospective design and the presence of mixed infections, these findings should be interpreted cautiously.

## Introduction

Necrotizing pneumonia (NP), also known as cavitary pneumonia or pulmonary cavitary necrosis, is a rare but severe complication of pediatric community-acquired pneumonia (CAP) (1, 2). Epidemiological data have shown a progressive increase in the incidence of NP between 1993 and 2004. Notably, French studies documented that NP cases constituted 4.5% of pediatric CAP hospitalizations from 2006 to 2009, with the incidence rising significantly to 9.0% from 2009 to 2011. Corresponding Asian studies (2015–2017) reported a prevalence of 2.3% among hospitalized patients with CAP (3). The diagnostic framework for NP remains poorly defined as it is primarily a histopathological condition. The pathological hallmarks of NP include inflammatory-mediated parenchymal destruction with subsequent liquefactive necrosis, frequently accompanied by vascular thrombosis and multifocal cavitation involving pulmonary segments or entire lobes (4). Radiological evaluation is the primary diagnostic modality, typically demonstrating parenchymal defects with characteristic thin-walled air- or fluid-filled cavities. Contrast-enhanced computed tomography (CT) revealed pathognomonic non-enhancing margins surrounding these lesions (5).

NP can be caused by diverse pathogens, with bacterial infections representing the predominant etiology. *S. pneumoniae* and *S. aureus* are the most frequently identified causative agents, as demonstrated by Chen et al. (6) and Sawicki et al. (2). In addition, other microorganisms, such as *Klebsiella pneumoniae*, *Streptococcus pyogenes*, *H. influenzae*, *Pseudomonas aeruginosa*, *Legionella pneumophila*, *Aspergillus species*, and *Mycobacterium tuberculosis* can also cause NP (1, 7–14). Emerging evidence highlights the role of viral pathogens in NP pathogenesis, particularly *respiratory syncytial virus*, *rhinovirus*, *adenovirus*, and *influenza virus (*2, 7, 15–17). Recent epidemiological trends demonstrate an increasing incidence of *M. pneumoniae*-associated NP (12, 18–21). Mixed infections, such as combinations of *S. pneumoniae* with *influenza virus* or *M. pneumoniae*, or *S. aureus* with *parainfluenza virus* are of particular clinical significance. These polymicrobial infections are consistently associated with more severe clinical presentations and poor patient outcomes than single-pathogen NP cases (22–25).

Chest imaging, particularly contrast-enhanced CT, is crucial for diagnosing NP because it provides direct visualization of the extent and severity of the lung damage (11, 26, 27). Currently, contrast-enhanced chest CT is the most sensitive imaging modality for NP diagnosis and can effectively differentiate NP from lung abscesses (12, 28). Imaging characteristics, including changes observed in static and portal venous phases and CT values, may exhibit variations that correlate with different causative pathogens. Furthermore, serum inflammatory markers such as white blood cell (WBC) count, C-reactive protein (CRP), procalcitonin (PCT), lactate dehydrogenase (LDH), and D-dimer exhibit pathogen-specific patterns and offer valuable diagnostic and prognostic information (29–32).

We conducted a retrospective study of 54 children diagnosed with NP at our hospital between January 2018 and January 2024. This design allowed a comprehensive evaluation of clinical, imaging, and inflammatory characteristics across pathogen types. We aimed to clarify the clinical features of NP and provide a scientific basis for improving diagnostic and therapeutic strategies.

## Materials and methods

### Patients

This single-center retrospective study included children diagnosed with NP between January 1, 2018, and January 31, 2024. Clinical data were extracted from patients' electronic medical records. Data collected included demographic characteristics, microbiological results, chest CT findings, serum inflammatory markers (white blood cell count [WBC], C-reactive protein [CRP], procalcitonin [PCT], lactate dehydrogenase [LDH], and D-dimer), and clinical outcomes. All NP diagnoses were confirmed based on chest CT findings. Although some patients initially underwent chest radiography during emergency evaluation, only CT findings were used for definitive diagnosis and included in this analysis.

### Inclusion criteria

Patients were eligible if they met all of the following criteria:

1. Age from 28 days to 18 years.
2. Clinical diagnosis of CAP.
3. Chest CT demonstrating features consistent with necrotizing pneumonia, characterized by multiple small air or fluid-filled cavities within areas of consolidation.
4. Availability of complete clinical and laboratory data.

### Exclusion criteria

Patients were excluded if any of the following conditions were present:

1. Clinical diagnosis of pulmonary abscess, defined radiologically as a single cavity with marginal enhancement.
2. Congenital lung cysts, pulmonary tuberculosis, or other cavitary lung diseases.
3. Underlying chronic conditions such as congenital heart disease, malignancy, or immunodeficiency.
4. Recurrent respiratory infections.
5. Absence of etiologic testing for respiratory viruses, Mycoplasma pneumoniae, or bacterial pathogens.

### Pathogen detection

All patients underwent polymerase chain reaction (PCR)–based fluorescent probe testing for common respiratory viruses, including respiratory syncytial virus, influenza A and B, parainfluenza virus, adenovirus, and rhinovirus (Sansure Biotech Inc., Changsha, China).

*Mycoplasma pneumoniae* (MP) infection was diagnosed using a combination of microbiological and serologic tests, including detection of *MP*-specific antibodies (IgM and/or IgG ≥1:160) (Mike Bio, Shenzhen, China) and/or *MP* nucleic acid detection (Tian Long Tech, Xi'an, China) in nasopharyngeal aspirates or bronchoalveolar lavage fluid. Serologic results were interpreted in conjunction with clinical findings and radiologic features to support the diagnosis of acute MP infection.

For suspected bacterial infections, metagenomic next-generation sequencing (mNGS) was performed on pleural effusion, blood, sputum, or bronchoalveolar lavage fluid samples by Chengdu Huayin Medical Laboratory (Chengdu, China) according to standard laboratory protocols and clinical indications. Because this was a retrospective study, not all patients underwent identical diagnostic testing, and pathogen identification relied on the available microbiological evidence combined with clinical correlation.

When multiple diagnostic results were available, pathogen identification was based on predefined microbiological criteria interpreted in conjunction with clinical findings. No single diagnostic method served as a gold standard.

### Pathogens not systematically evaluated

The present study focused on bacterial pathogens, common respiratory viruses, and *Mycoplasma pneumoniae* routinely tested in our institution.Other potential respiratory pathogens, including fungal infections, atypical bacteria such as *Chlamydia pneumoniae*, and less common viral pathogens not included in the PCR panel, were not systematically evaluated and therefore were not included in pathogen-specific analyses.

### Pathogen classification and management of mixed infections

Patients were stratified into pathogen-specific groups according to microbiological findings. Cases in which a single pathogen was identified were assigned to the corresponding pathogen group for subsequent comparative analyses.

Patients with evidence of mixed-infection (detection of more than one pathogen) were analysed separately in descriptive analyses. To minimise potential classification bias, these cases were excluded from pathogen-specific inferential statistical comparisons evaluating differences in imaging characteristics and inflammatory biomarkers among single-pathogen groups.

Mixed infection cases were not reassigned to any single-pathogen category but were retained in analyses describing the overall clinical characteristics of necrotizing pneumonia.

### Serum inflammatory markers

Serum markers, including WBC count, CRP, PCT, LDH, and D-dimer, were measured within 3 days of admission. In cases where multiple tests were performed, the highest values were recorded as baseline data.

### Contrast-Enhanced chest CT protocol

NP was diagnosed based on contrast-enhanced chest CT findings demonstrating areas of parenchymal consolidation with internal low-attenuation regions suggestive of necrosis and cavitary change. In this study, two representative components within the lesions were analyzed: air-containing cavitary regions and solid consolidation components.

Chest CT was performed in children with suspected complicated pneumonia when there was poor clinical response after 5–7 days of appropriate antibiotic therapy, clinical deterioration including persistent high fever or worsening respiratory symptoms, or abnormal chest radiographs suggestive of necrosis or abscess formation. The timing of CT scans ranged from 6 to 18 days after symptom onset, with a median of 9 days, reflecting the evolving pathological process of NP.

Fifty-three patients underwent contrast-enhanced chest CT within 48 h of admission using standardized pediatric imaging protocols (120 kVp with automated tube current modulation). Imaging acquisitions included non-contrast, arterial phase (30–35 s delay), and portal venous phase (60–70 s delay) sequences.

CT attenuation values (Hounsfield Units, HU) of cavitary regions and solid consolidation components were measured using a standardized region-of-interest (ROI) protocol. Circular ROIs were manually placed within representative cavitary regions and solid consolidation components of the lesions while carefully avoiding adjacent vessels, bronchi, pleural fluid, and beam-hardening artifacts. ROI size was adjusted according to lesion dimensions to ensure representative sampling. For each lesion, three ROIs were placed at separate representative levels, and the final attenuation value was calculated as the mean of these measurements. When multiple lesions were present, the largest representative lesion was selected for analysis to ensure measurement consistency and minimize sampling variability across heterogeneous lesions. The imaging phase designation (arterial or portal venous phase) was determined according to the scanning protocol and verified using DICOM acquisition metadata.

All CT images were independently reviewed by two board-certified pediatric radiologists with more than 10 years of thoracic imaging experience, who were blinded to clinical and microbiological information. Discrepancies were resolved by consensus.

### Radiation exposure

Radiation doses were maintained within pediatric reference levels recommended by the American College of Radiology (2022). Age-adjusted computed tomography dose index (CTDIvol) and dose-length product (DLP) ranges were as follows:

Computed Tomography Dose Index: 0–1 month: 1.6 mGy; 1 month to 4 years: 1.6–2.4 mGy; 4–10 years: 2.4–2.9 mGy; 10–14 years: 7.2 mGy; 14–18 years: 7.2–14 mGy.

Dose-Length Product (DLP): 0–1 month: 0 mGy·cm; 1 month to 4 years: 31–58 mGy·cm; 4–10 years: 58–95 mGy·cm; 10–14 years: 272 mGy·cm; 14–18 years: 272–596 mGy·cm.

Radiation exposure was kept within these recommended limits to ensure patient safety while maintaining diagnostic accuracy.

One patient (Figure 6) underwent an initial non-contrast CT followed by a repeat contrast-enhanced CT one month later, which showed marked resolution of pulmonary lesions. Congenital pulmonary anomalies (including pulmonary cysts, congenital pulmonary airway malformation, and pulmonary sequestration) were excluded. The cumulative radiation exposure in this patient remained within established pediatric safety thresholds (first scan: DLP 56, effective dose 2.18 mSv; second scan: DLP 61, effective dose 2.38 mSv).

### Statistical analysis

Statistical analyses were performed using SPSS version 25.0 (IBM Corp., Armonk, NY, USA). Comparative analyses of biomarkers and imaging characteristics were conducted only among single-pathogen groups to minimize classification bias. Given the limited sample sizes in several pathogen subgroups, these analyses were considered exploratory. Normality of continuous variables was assessed using the Shapiro–Wilk test. Normally distributed data are presented as mean ± standard deviation (SD) and were compared using independent-sample t-tests for two-group comparisons or one-way analysis of variance (ANOVA) for comparisons among multiple groups, followed by Bonferroni correction for *post hoc* comparisons when analysing biomarkers across different pathogen groups. Non-normally distributed data are expressed as median (interquartile range) [M (P25–P75)] and were analysed using the Mann–Whitney U test for two-group comparisons or the Kruskal–Wallis test with Dunn–Bonferroni *post hoc* correction for multiple-group comparisons. Categorical variables were compared using the chi-square (*χ*^2^) test or Fisher's exact test when appropriate. Correlation analyses were performed using Spearman's rank correlation coefficient because of the small sample size and the potential non-normal distribution of the data. Logistic regression analysis was performed to explore potential associations between imaging parameters and pathogen groups; however, because of the limited cohort size and the low number of events per variable, these analyses were interpreted as exploratory and were not intended for predictive modelling. Receiver operating characteristic (ROC) curve analysis was not performed because the primary aim of this study was descriptive characterization of imaging patterns rather than development of diagnostic prediction models, and the small subgroup sizes would limit the reliability of such analyses. However, because of the limited cohort size and the low number of events per variable, the regression analyses were interpreted as exploratory and were not intended for predictive modelling. A two-tailed *p*-value < 0.05 was considered statistically significant.

Interobserver agreement for quantitative CT attenuation measurements was evaluated using intraclass correlation coefficients (ICCs). Agreement for categorical imaging features was assessed using Cohen's kappa statistics.

## Results

This study included 54 pediatric patients (29 males, 25 females; mean age 6.29 ± 3.68 years). Microbial profiling identified single-pathogen infections in 45 cases and mixed infections in 9 cases. For pathogen-specific comparative analyses, only single-pathogen cases were included to minimize potential classification bias, while mixed infections were analysed descriptively. Among single-pathogen cases, age-stratified differences in pathogen distribution were observed (*χ*^2^ = 18.7, *p* = 0.004). Because of the very small number of cases for certain pathogens (e.g., *H. influenzae* and *adenovirus*), findings for these subgroups should be interpreted as descriptive observations rather than definitive pathogen-specific patterns. Bacterial pathogens were more frequently identified in children under 3 years of age, particularly S. aureus (5/8, 62.5%) and H. influenzae (2/8, 25%), and were associated with higher rates of systemic complications such as sepsis (5/7, 71.4%) and toxic encephalopathy (3/7, 42.9%). In contrast, M. pneumoniae predominated in older children (3–6 years: 14/30, 46.7%; ≥ 6 years: 9/14, 64.3%) and was more frequently associated with respiratory complications, including pleural effusion (16/30, 53.3%) and respiratory failure (12/30, 40%), as well as dyspnea (11/14, 78.6%) and hemoptysis (3/14, 21.4%).

Correlation analysis among single-pathogen cases demonstrated a strong negative association between bacterial infection and age (Spearman *ρ* = −0.82, *p* < 0.001) and a positive association between M. pneumoniae infection and age (Spearman *ρ* = 0.79, *p* < 0.001). Increasing age was inversely associated with the risk of systemic complications (OR 0.54 per year increase, 95% CI 0.38–0.72). Given the relatively small sample size, this association should be interpreted with caution.

**Figure 1:**
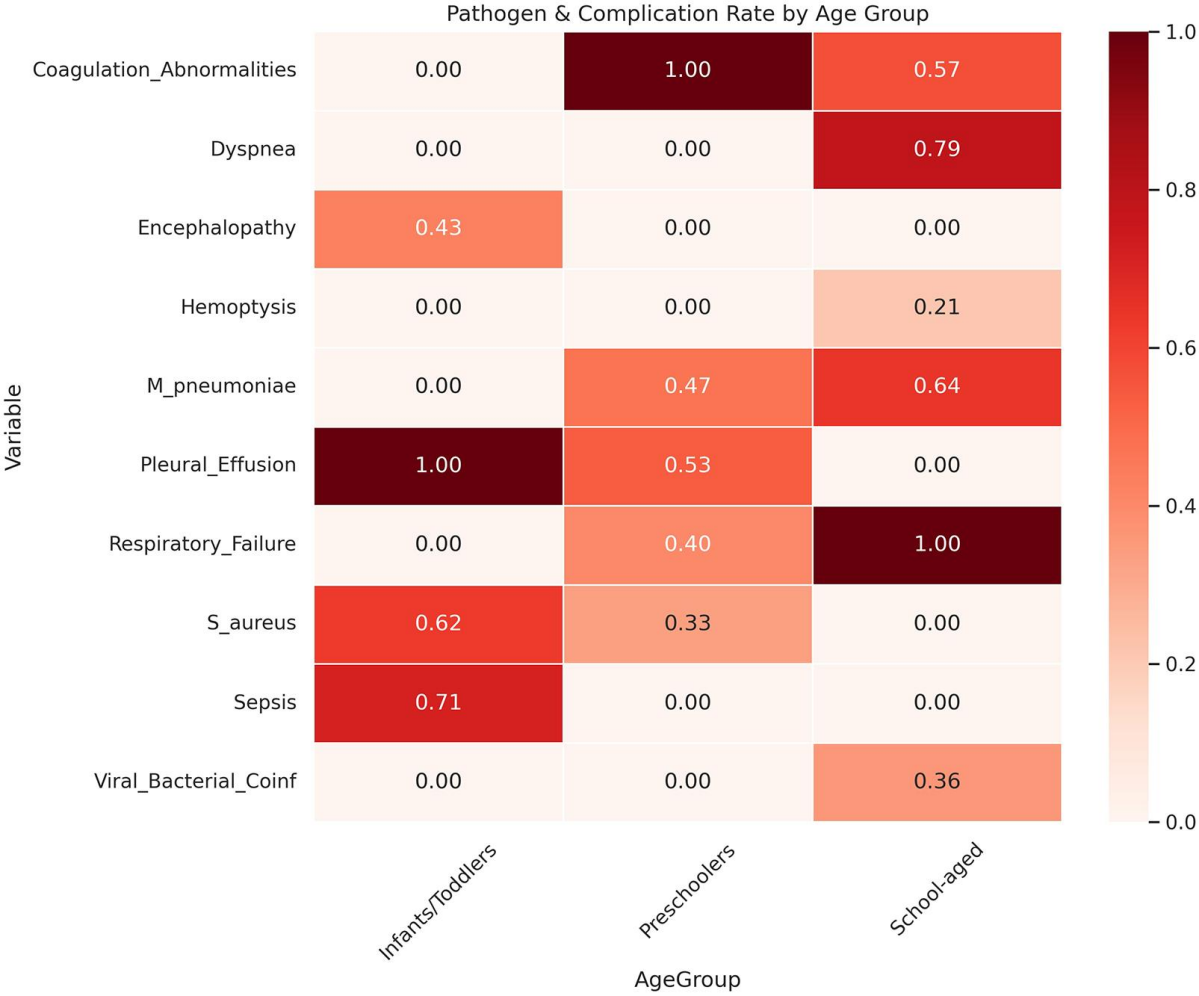
Heat map of pathogen distribution and incidence of major complications in different age groups.

**Figure 2:**
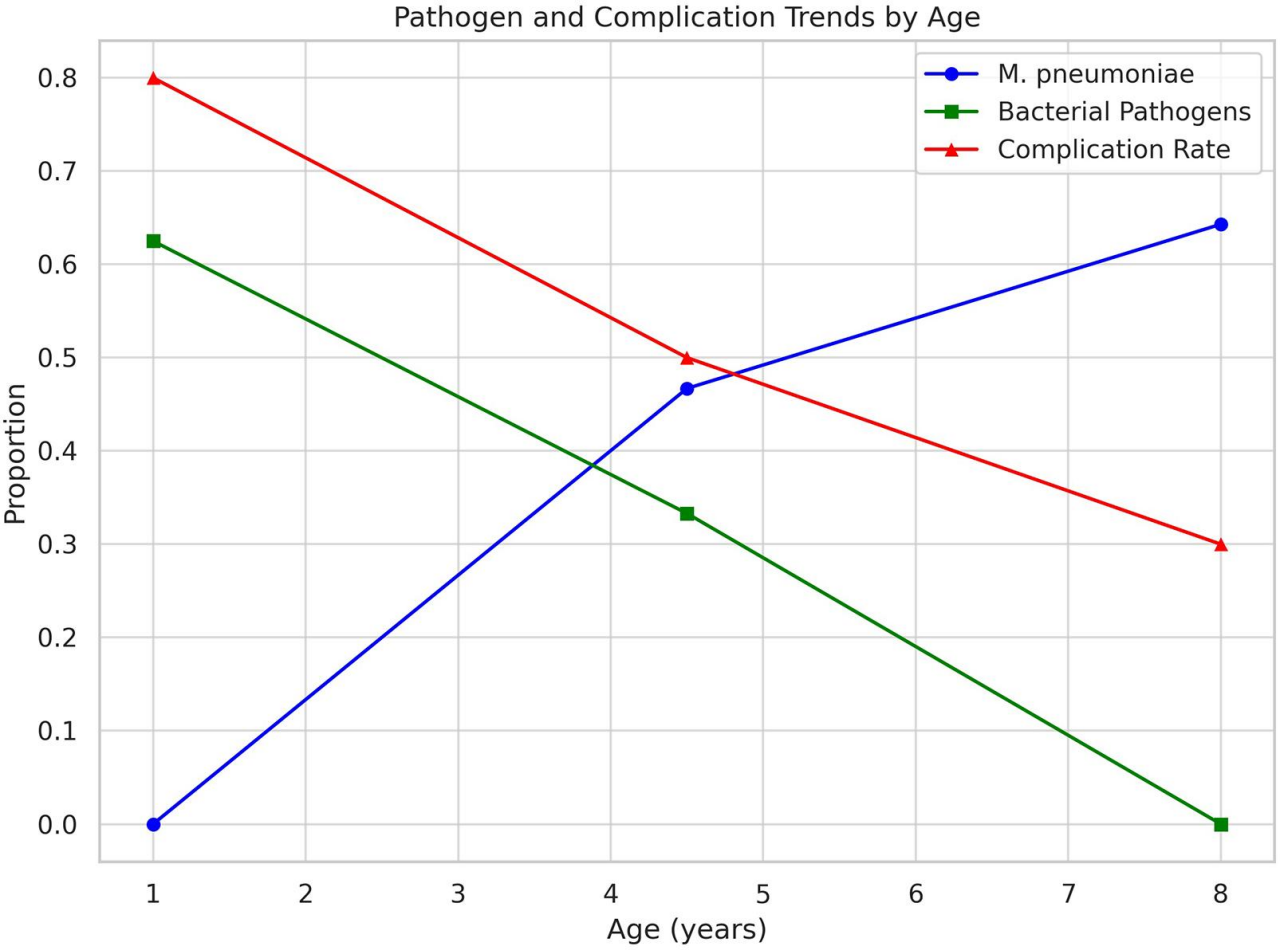
Trend_pathogen_complications by age.

Mixed infections (*n* = 9) most frequently involved *M. pneumoniae* coexisting with *S. aureus*, *S. pneumoniae*, or influenza A virus (Tables 1 and 2, Figures 1 and 2).

**Table 1:** Age-stratified clinical characteristics, predominant pathogens, and major complications.

Age group	Sex, *n* (%)	Predominant pathogens	Clinical features	Major complications
Infants/Toddlers <3 years	M: 8 (14.8) F: 3 (5.6)	*SA* (62.5%) *HI* (25%)	Rapidly progressive febrile cough	Sepsis (71.4%) Toxic encephalopathy (42.9%) Pleural effusion
Preschoolers 3–6 years	M: 8 (14.8) F: 7 (12.9)	*MP* (46.7%) *SA* co-infection (33.3%)	Persistent febrile cough	Pleural effusion (53.3%) Respiratory failure (40%) Coagulation abnormalities
School-aged ≥6 years	M:13 (24.1) F: 15 (27.8)	*MP* predominance (64.3%) Viral–bacterial co-infection (35.7%)	Dyspnea (78.6%) Hemoptysis (21.4%)	Coagulation abnormalities (57.1%) Respiratory failure

**Table 2:** Distribution of pathogens in 54 children with necrotizing pneumonia.

Pathogen(s)	*n* (%)	Diagnostic Method(s)
*SA*	8 (14.8)	Sputum culture; blood culture; BALF metagenomic sequencing
*SP*	5 (9.3)	Sputum culture; BALF metagenomic sequencing
*MP*	29 (53.7)	Throat swab; serum antibody titer; BALF metagenomic sequencing
*Adenovirus*	1 (1.9)	Throat swab; BALF metagenomic sequencing
*HI*	2 (3.7)	Sputum culture; BALF metagenomic sequencing
*MP* *+* *SA*	2 (3.7)	Serum antibody titer; BALF metagenomic sequencing
*MP* *+* *SP*	3 (5.6)	Sputum culture; serum antibody titer; BALF metagenomic sequencing
*HI* *+* *IBV*	1 (1.9)	Sputum culture; antigen detection
*MP* *+* *HI* *+* *IBV*	1 (1.9)	Sputum culture; serum antibody titer; BALF metagenomic sequencing
*MP* *+* *SA* *+* *SP*	1 (1.9)	Serum antibody titer; BALF metagenomic sequencing
*MP* *+* *IAV*	1 (1.9)	Throat swab; antigen detection; BALF metagenomic sequencing

Among the 54 pediatric patients, *M. pneumoniae* was the most frequently detected pathogen, identified in 36 of 54 cases (66.7%), including 28 cases of isolated MP infection and 8 mixed infections involving MP. Mixed infections were identified in 8 of 54 patients (14.8%), most commonly involving *M. pneumoniae* in combination with bacterial pathogens such as *S. aureus*, *S. pneumoniae*, or *H. influenzae*, and occasionally with respiratory viruses. Multiple diagnostic methods were employed, including throat swabs, sputum and blood cultures, serum antibody testing, antigen detection, and metagenomic sequencing of bronchoalveolar lavage fluid (BALF). Among these methods, BALF metagenomic sequencing played a key role in pathogen identification, particularly in complex or mixed infections (Table 2).

Serum Inflammatory Markers and CT Values by Pathogen are as follows:

*S. aureus* (*n* = 8): WBC: 13.43 (5.07–20.01), CRP: 140 (71.8–200), PCT: 0.675 (0.17–4.45), LDH: 321.1 (268.95–355.3), and D-dimer: 3.45 (0.59–4.45). CT attenuation values of cavitary regions: Pre-contrast: −740 to −763, Arterial phase: −604 to −688, and Portal phase: −701 to −737. Solid consolidation components: Pre-contrast: 29–35, Arterial phase: 38–57, and Portal phase: 62–70.

*S. pneumonia* (*n* = 5): WBC: 13.89 (8.41–21.63), CRP: 20.5 (8–200), PCT: 0.22 (0.09–35.4), LDH: 244.8 (221.3–607.4), and D-dimer: 1.5 (0.31–3.87). CT attenuation values of cavitary regions: Pre-contrast: −706 to −779, Arterial phase: −728 to −756, and Portal phase: −654 to −665. Solid consolidation components: Pre-contrast: 1–7, Arterial phase: 49–60, Portal phase: 105–140.

*M. pneumoniae* (*n* = 28): WBC: 9.9 (7.65–12.19), CRP: 36 (11.5–85.64), PCT: 0.16 (0.15–0.31), LDH: 320.5 (226.7–431.8), and D-dimer: 2.22 (0.65–5.54). CT attenuation values of cavitary regions: Pre-contrast: −765 to −685, Arterial phase: −622 to −688, and Portal phase: −683 to −770. Solid consolidation components: Pre-contrast: 22–31, Arterial phase: 26–30, Portal phase: 23–29.

A comparison of serum inflammatory markers between the three most common pathogens revealed a statistically significant difference in CRP levels (*p* < 0.05). However, no significant differences were observed for WBC, PCT, or D-dimer among the groups (*p* > 0.05).

### Infectious etiology, D dimer, PCT, LDH

Table 3 lists the three most common pathogens and serum inflammatory markers found in children diagnosed with NP. In our research, *M. pneumoniae* and *S. aureus* emerged as the top two pathogens associated with NP, with *S.pneumonia* being the next most prevalent.

**Table 3:** Serum inflammation indexes of necrotizing pneumonia caused by different pathogens; *P* < 0.05 was statistically significant.

Variable	*SA* (*n* = 8)	*SP* (*n* = 5)	*MP* (*n* = 29)	*P*-value
WBC (×10⁹/L)	13.43 (5.07–20.01)	13.89 (8.41–21.63)	9.90 (7.65–12.19)	0.455
CRP (mg/L)	140 (71.8–200)	20.5 (8–200)	36 (11.5–85.64)	0.032
D-dimer (µg/mL)	3.45 (0.59–4.45)	1.50 (0.31–3.87)	2.22 (0.65–5.54)	0.161
PCT (ng/mL)	0.675 (0.17–4.45)	0.22 (0.095–35.4)	0.16 (0.05–0.31)	0.457
LDH (U/L)	321.1 (268.95–355.3)	244.8 (221.3–607.4)	320.5 (226.7–431.8)	0.967

### Enhanced chest CT findings

**Figure 3:**
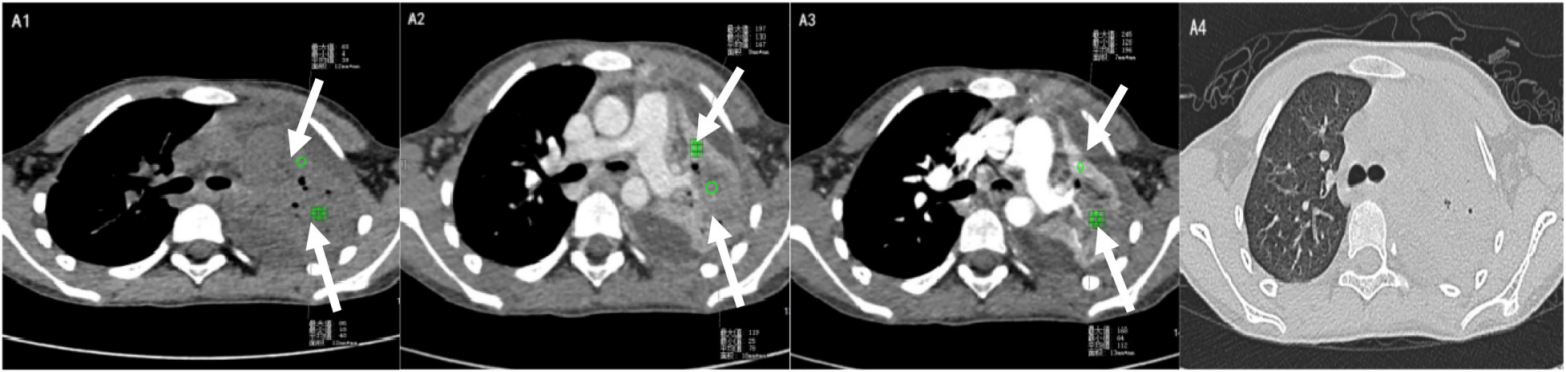
*SA.* A1: Plain CT scan showing multilobar consolidation with internal low-attenuation areas; A2: Portal venous phase demonstrating peripheral rim enhancement; A3: Arterial phase revealing heterogeneous enhancement; A4: Lung window illustrating thin-walled cavitation (<2 mm).

*S. aureus* NP typically presents with multilobar consolidation, predominantly in the lower lobes, containing multiple internal low-attenuation areas (Table 4). Thin-walled cavities (<2 mm) with possible air–fluid levels are frequently observed, and complications such as empyema and bronchopleural fistula are common (Figure 3).

**Table 4:** CT attenuation values of cavitary and solid components in single-pathogen infection.

CT Phase	SA	SP	MP	Adenovirus	HI
Air-containing regions (HU)					
Non-contrast	−740 to −763	−706 to −779	−765 to −685	−717 to −735	−607 to −676
Arterial phase	−604 to −688	−728 to −756	−622 to −688	−524 to −604	−592 to −618
Portal venous phase	−701 to −737	−654 to −665	−683 to −770	−517 to −592	−593 to −643
Solid consolidation regions(HU)					
Non-contrast	29 to 35	1 to 7	22 to 31	12 to 15	26 to 33
Arterial phase	38 to 57	49 to 60	26 to 30	21 to 31	23 to 30
Portal venous phase	62 to 70	105 to 140	23 to 29	32 to 38	27 to 33
Cavitation	Yes	No	No	No	No

**Figure 4:**
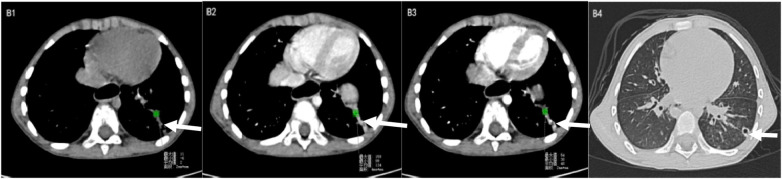
*SP.* B1: Plain CT scan showing focal upper-lobe consolidation with an internal low-attenuation area; B2: Portal venous phase demonstrating peripheral rim enhancement; B3: Arterial phase revealing heterogeneous enhancement; B4: Lung window illustrating a round thick-walled cavity.

*S.pneumonia* NP typically presents with lobar consolidation, most commonly involving the upper lobes, with a single thick-walled cavity (>4 mm wall thickness) and central low-attenuation areas (Table 4). Air–fluid levels are usually absent. Pleural thickening is common, whereas empyema is relatively rare (Figure 4).

**Figure 5:**
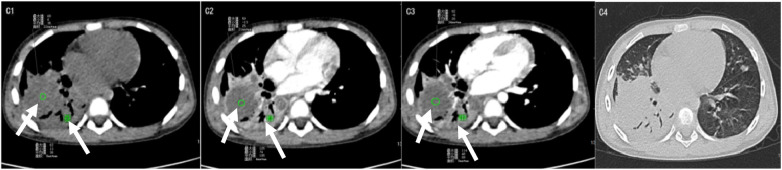
*MP.*C1: plain CT scan showing patchy ground-glass opacities with segmental consolidation; C2: portal venous phase demonstrating mild heterogeneous enhancement; C3: arterial phase revealing mild enhancement; C4: lung window illustrating air bronchograms without conspicuous cavitation.

*M. pneumoniae* NP typically presents with patchy ground-glass opacities and segmental consolidation, with small internal low-attenuation areas within the consolidated regions (Table 4). Cavitation is uncommon. Lesions are usually confined to a single lobe and frequently show air bronchograms. Pleural involvement is minimal, occasionally accompanied by small pleural effusions (Figure 5).

**Figure 6:**
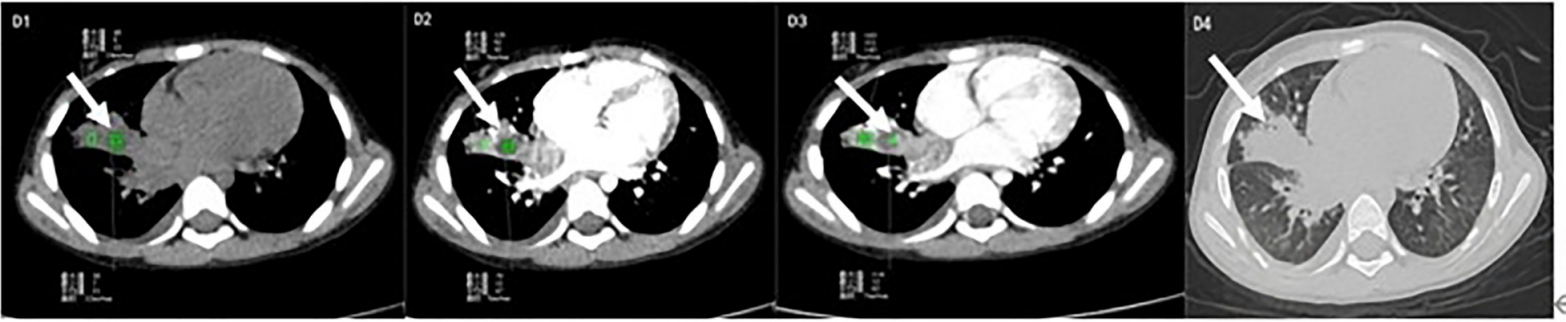
Adenovirus. D1: plain CT scan showing multifocal consolidation with extensive ground-glass opacities; D2: portal venous phase demonstrating heterogeneous enhancement; D3: arterial phase revealing mild peripheral enhancement; D4: lung window illustrating focal consolidation with surrounding ground-glass opacities.

*Adenoviral* NP typically demonstrates multifocal consolidations with extensive ground-glass opacities, often showing a “patchy-map” distribution (Table 4 and Figure 6).

**Figure 7:**
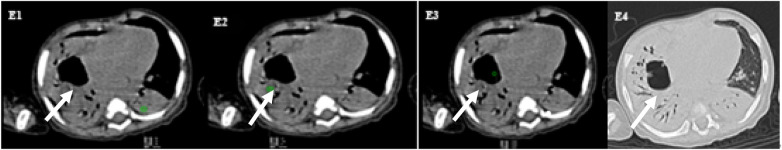
*HI.* E1: Plain CT scan showing right lower-lobe consolidation with internal low-attenuation foci and cavitation; E2: Portal venous phase demonstrating heterogeneous enhancement; E3: Arterial phase revealing similar enhancement pattern; E4: Lung window illustrating clustered microcavities and thin-walled cavitation.

*H.influenzae* NP typically demonstrates right lower lobe consolidation with multiple internal low-attenuation foci (Table 4) and clustered microcavities measuring approximately 4–6 mm. Adjacent interlobular septal thickening and small pleural effusion may also be present (Figure 7).

**Figure 8:**
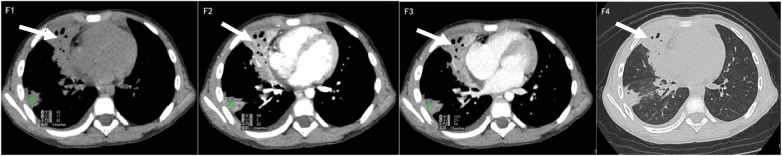
F1: plain CT scan showing lobar consolidation with central low-attenuation areas; F2: portal venous phase demonstrating peripheral enhancement; F3: arterial phase revealing heterogeneous enhancement; F4: lung window illustrating multiple thin-walled cavities.

**Figure 9:**
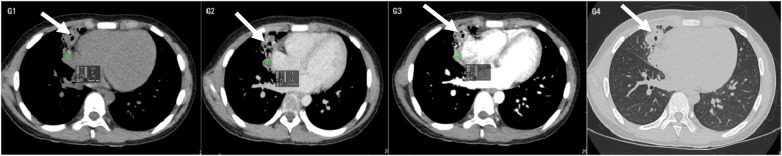
Mixed infection (*MP* *+* *SP*). G1: Plain CT scan showing lobar consolidation with central low-attenuation areas and cavitation; G2: Portal venous phase demonstrating heterogeneous enhancement; G3: Arterial phase revealing early peripheral enhancement; G4: Lung window illustrating a round thick-walled cavity.

**Figure 10:**
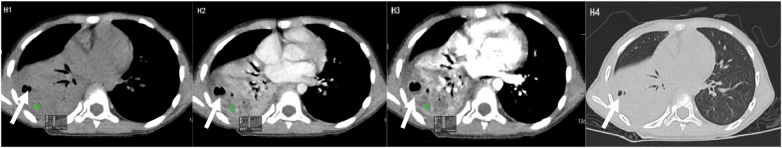
Mixed infection (*MP* *+* *SA* *+* *SP*)*.* H1: Plain CT scan showing extensive consolidation with irregular cavitation; H2: Portal venous phase demonstrating heterogeneous enhancement; H3: Arterial phase revealing peripheral enhancement; H4: Lung window illustrating irregular thin-walled cavitation.

**Figure 11:**
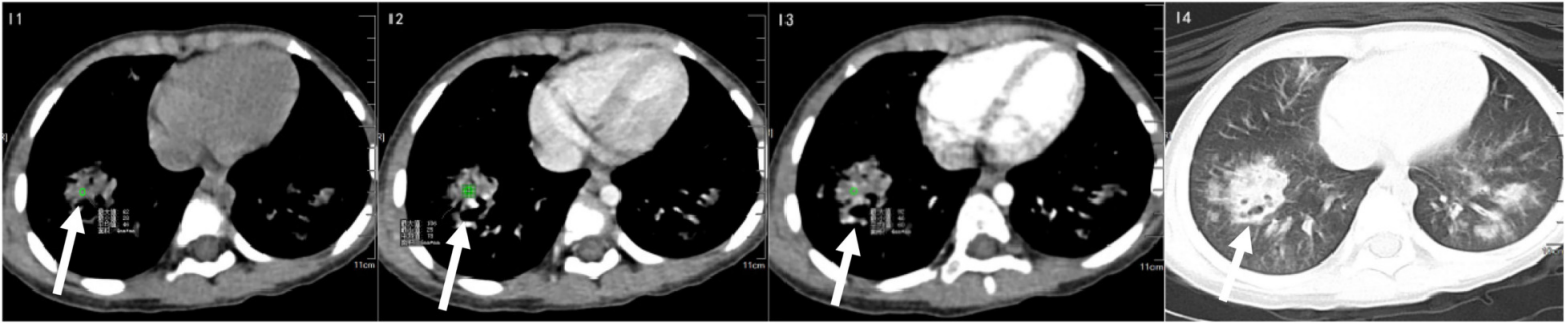
Mixed infection (*MP* *+* *HI* *+* *IBV*). I1: Plain CT scan showing focal consolidation with central cavitation; I2: Portal venous phase demonstrating heterogeneous enhancement; I3: Arterial phase revealing peripheral enhancement; I4: Lung window illustrating cavitation with scattered bilateral ground-glass opacities.

**Figure 12:**
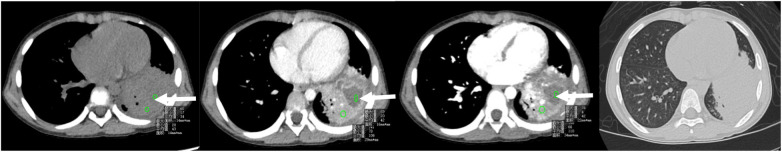
Mixed infection (*MP* *+* *IAV*)*.* J1: Plain CT scan showing patchy consolidation with internal low-attenuation areas and scattered thin-walled cavities; J2: Portal venous phase demonstrating heterogeneous enhancement; J3: Arterial phase revealing peripheral enhancement; J4: Lung window confirming thin-walled cavitation.

On contrast-enhanced chest CT, mixed-infection cases demonstrated imaging features that overlapped with patterns observed in single-pathogen groups (Table 5). These findings included multifocal consolidations with lobar or peribronchial distributions, heterogeneous enhancement, low-attenuation areas (Figures 8–12), and ground-glass opacities. Cavitary changes with variable morphology were observed in some cases, including thick-walled cavities and clustered microcavities. Air–fluid levels and internal debris were occasionally noted. Owing to the limited number of mixed-infection cases, these findings are presented descriptively without formal statistical comparison.

**Table 5:** CT attenuation values of cavitary and solid components in mixed-pathogen infection.

CT Phase	MP + SA	MP + SP	MP + SA + SP	MP + HI + IBV	MP + IAV
Air-containing regions (HU)					
Non-contrast	42–47	42–46	47–52	43–46	35–40
Arterial phase	122–133	80–123	143–162	60–65	163–168
Portal venous phase	106–115	58–78	107–111	78–80	113–142
Solid consolidation regions (HU)					
Non-contrast	32–41	30–52	34–36	−900 – −910	33–36
Arterial phase	62–80	65–81	36–39	—	49–53
Portal venous phase	75–82	61–72	43–48	—	42–48
Cavitation	Yes	Yes	Yes	Yes	Yes

HU, Hounsfield Units; —: Not available.

The interobserver agreement for CT attenuation measurements was excellent (ICC = 0.87, 95% CI 0.81–0.92). Cohen's *κ* values for categorical imaging features ranged from 0.64 to 0.82.

## Discussion

Necrotizing pneumonia (NP) is a severe complication of pediatric pneumonia that has been increasingly recognized in recent years (33). In this study, we analyzed the clinical, laboratory, and imaging characteristics of pediatric NP by integrating microbiological findings with radiologic and inflammatory profiles.

Consistent with previous reports, *S. aureus*, *S. pneumoniae*, and *M. pneumoniae* were the most frequently identified pathogens in our cohort (17, 32, 34). An age-related distribution pattern was observed, with bacterial pathogens more commonly identified in younger children and *M. pneumoniae* predominating in older age groups. This pattern is consistent with the well-established epidemiology of pediatric respiratory infections and reflects the increasing recognition of *M. pneumoniae* as an important etiological agent of NP. However, given the presence of mixed infections and the limited sample size, these age–pathogen associations should be interpreted cautiously. The biological mechanisms underlying age-dependent susceptibility require further investigation.

Fever and cough were the most common clinical manifestations, whereas older children more frequently presented with chest pain, dyspnea, and hemoptysis (35). A substantial proportion of patients developed systemic complications, reflecting the severity of disease progression. Elevated inflammatory markers, persistent fever, and pleural involvement were commonly observed in NP and may serve as clinical warning signs. Although CRP levels appeared higher in *S. aureus* infections compared with *M. pneumoniae* infections, these differences represent associations rather than definitive pathogen-specific markers and should be interpreted cautiously without prospective validation.

Chest CT played a central role in the diagnosis and clinical evaluation of NP, demonstrating characteristic findings such as lung consolidation, cavitation, and complications including bronchopleural fistula, pleural effusion, and empyema (36). Variations in CT attenuation values were observed among pathogen groups. S. aureus infections tended to demonstrate more extensive cavitary changes within areas of consolidation and were frequently accompanied by empyema or pyopneumothorax. *S. pneumoniae* cases showed more localized cavitation, whereas *M. pneumoniae* infections often demonstrated more variable radiologic patterns. Attenuation values across pathogen groups showed relatively wide ranges, which likely reflect the heterogeneous composition of necrotizing pneumonia lesions, including areas of consolidation, necrotic tissue, cavitation, inflammatory exudates, and partial aeration. In particular, air-containing cavitary components may produce markedly negative attenuation values approaching those of air, whereas areas of consolidation and inflammatory exudate demonstrate higher attenuation values. In addition, differences in ROI placement and variations in lesion evolution at different stages of disease may contribute to attenuation variability. These findings should therefore be interpreted as descriptive imaging observations rather than pathogen-specific diagnostic indicators. Given the retrospective study design and the presence of mixed infections, further prospective studies are needed to determine whether CT attenuation values have reproducible diagnostic or prognostic value. In addition, the timing of CT examinations was determined by clinical indications rather than a standardized protocol, which may have contributed to variability in imaging findings.

No deaths occurred in our cohort; however, NP remains a potentially severe condition. Early diagnosis and timely intervention are critical to reducing complications and improving prognosis. With the increasing recognition of *M. pneumoniae* as a causative agent of NP, particularly in older children with progressive symptoms and atypical imaging findings, clinicians should maintain a high index of suspicion for NP in such cases.

This study provides a comprehensive description of the clinical, laboratory, and imaging characteristics of pediatric necrotizing pneumonia. Chest CT remains an important tool for diagnosis and clinical assessment, and the imaging patterns observed across pathogen groups may help clinicians in differential evaluation and disease monitoring.

### Limitations

Several limitations should be considered. First, this was a retrospective, single-center study, which limits generalizability and precludes causal inference. The overall sample size was modest, and certain pathogen subgroups (e.g., *Streptococcus pneumoniae* and *adenovirus*) included relatively few patients, reducing statistical power. Mixed infections were analyzed descriptively and were excluded from comparative analyses, which may influence the interpretation of pathogen-specific patterns.

Pathogen identification was based on multiple diagnostic modalities, including PCR-based viral detection, serologic and nucleic acid testing for *M. pneumoniae*, and mNGS for suspected bacterial infections. These methods have different sensitivity, specificity, and temporal characteristics. In particular, not all patients underwent uniform diagnostic testing (e.g., mNGS), and pathogen classification relied on available microbiological results combined with clinical correlation. The use of serologic criteria for *M. pneumoniae* (e.g., IgG ≥1:160) may also carry a risk of misclassification between past and acute infection.

The discriminative performance of CT attenuation values was not formally evaluated using receiver operating characteristic (ROC) analysis or multivariable modelling. Therefore, the observed attenuation differences should be interpreted as descriptive associations rather than validated diagnostic markers. In addition, CT attenuation measurements were obtained using manually placed circular regions of interest (ROIs), which may introduce measurement variability despite standardized measurement protocols.

Finally, outcome-based validation was not performed. Associations between imaging findings and clinical endpoints (e.g., length of hospital stay, complication severity, or long-term sequelae) were not examined, and treatment strategies were not analyzed according to pathogen subgroup. Moreover, the timing of CT examinations varied according to clinical indications rather than a standardized imaging schedule, which may introduce variability related to different stages of disease evolution.

Prospective, multicenter studies with standardized imaging timing and pathogen detection protocols are warranted to confirm these findings and clarify their clinical relevance.

## Conclusion

NP is an uncommon but potentially severe complication of community-acquired pneumonia in children, often presenting with abrupt clinical deterioration, systemic inflammatory manifestations, and persistent fever. Although fever and cough are the most common initial symptoms, older children more frequently exhibit dyspnea, pleuritic chest pain, and hemoptysis. Age-related differences in pathogen distribution were observed, and variations in clinical features, inflammatory markers, and CT findings appeared to be associated with different pathogen groups. These observations should be interpreted cautiously given the retrospective design and the presence of mixed infections. Early recognition, appropriate antimicrobial therapy, and careful clinical monitoring remain essential for improving outcomes in pediatric NP.

## Data Availability

The original contributions presented in the study are included in the article/Supplementary Material, further inquiries can be directed to the corresponding author.
